# Agency and Communion in Narratives of Older Adults With and Without Mild Cognitive Impairment

**DOI:** 10.1093/geroni/igaf122.4064

**Published:** 2025-12-31

**Authors:** Xiangyu (Annie) Pei, Emily Tso, Gelila Ambellu, Da Eun Kim, Hsiao-Wen Liao, Emily Mroz

**Affiliations:** Emory University, Atlanta, Georgia, United States; Emory University, Atlanta, Georgia, United States; Emory University, Atlanta, Georgia, United States; Georgia Institute of Technology, Atlanta, Georgia, United States; Georgia Institute of Technology, Atlanta, Georgia, United States; Emory University, Atlanta, Georgia, United States

## Abstract

Older adults with mild cognitive impairment (MCI) tend to worry about losing their memories and sense of identity, putting them at risk of reduced wellbeing. The retrieval of autobiographical memories (AM) about positive life events predicts greater life satisfaction and positive self-conceptions of future selves in adults. Therefore, autobiographical memory interventions for people with cognitive change have grown in popularity. However, much existing research about AM has focused on dementia rather than MCI, and technology-assisted memory sharing remains empirically underexplored. This study examined agency and communion – two features linked to wellbeing in late life – in AM elicited from adults with MCI (n = 17) and those without MCI (n = 18). We examined whether technology-enhanced memory sharing (i.e., photo memory viewing in virtual reality (VR) versus positive memory outside VR) influences narration of agency and communion. Transcripts were coded by two coders (kappa = 1.0). Results demonstrated that communion and agency appeared across both groups and conditions. Agency did not differ significantly between both groups and conditions. Communion in non-VR assisted positive memories was higher among adults without MCI (M = 1.00) than those with MCI (M = .29; p = .034). However, both groups included similar amounts of communion in VR-assisted photo memories. During this presentation, we will discuss our protocols and preliminary takeaways, including that the VR-assisted photo memory task may support people with MCI in sharing more communal narratives. Further research can explore how VR environments promote rich memory sharing in different older adult populations.

